# Adjusting health care: practicing care for socially vulnerable type 2 diabetes patients

**DOI:** 10.1186/s12913-021-06964-6

**Published:** 2021-09-10

**Authors:** Sofie á Rogvi, Ann Dorrit Guassora, Gitte Wind, Nina Tvistholm, Solveig May-Britt Jansen, Inge Birgitte Hansen, Hans Joergen Duckert Perrild, Ulla Christensen

**Affiliations:** 1 Department of Public Health, Section of Social Medicine, University of Copenhagen, Øster Farimagsgade 5A, 1014 Copenhagen K, Denmark; 2 Department of Public Health, Section of General Practice, University of Copenhagen, Copenhagen, Denmark; 3 Department of Nursing and Nutrition, University College Copenhagen, Copenhagen, Denmark; 4 Department of Endocrinology, Bispebjerg Hospital, Copenhagen, Denmark

## Abstract

**Background:**

Type 2 diabetes cluster in lower social groups and people with type 2 diabetes from lower social groups experience more complications, benefit less from health services and live shorter lives than people with type 2 diabetes from higher social groups. Different logics govern diabetes care and potentially influence the possibility of socially vulnerable type 2 diabetes patients to access and benefit from health services. In order to understand which practice and underlying logic enable socially vulnerable type 2 diabetes patients to access and benefit from diabetes care we aim to describe what professionals at a specialized diabetes clinic do to adjust services to patient’s needs and make the tasks involved in diabetes care doable for socially vulnerable patients and how this work is embedded in an organizational and moral context.

**Methods:**

Ethnographic fieldwork combining participant observation and interviews was carried out between February 2017 and March 2018 in a specialized diabetes clinic located in a socially deprived area in the capital region of Denmark. Sixteen patients (9 male, 7 female, aged 35-73 years) and 12 professionals (7 doctors, 4 nurses, 1 secretary) participated in the study. We used Annemarie Mol’s concept of “the logic of care” to guide our analysis.

**Results:**

Our analysis shows that the logic of care and the care practices in this clinic are characterized by a needs-based approach to treatment involving adjustment of services (permeability, timing, and content) and seeing the patient as a person with many needs. Throughout our description of selected care practices, we both characterize how health professionals practice this particular logic of care and the organizational and normative conditions that this logic is entangled with.

**Conclusions:**

Practicing diabetes care based on patients’ needs involves individualization, something often described as an element of patient centred care. Our study shows that this ideal of individualization and adjustment of treatment is possible in practice. Organizational flexibility and an organizational culture that values patient needs enable needs-based care. In order for socially vulnerable type 2 diabetes patients to benefit from health services it is necessary to create conditions under which professionals can attend to these patients’ multiple and complex needs. Adjusting care to these needs demand a variety of professional efforts some of which are hardly predictable or standardisable.

## Introduction

The number of people with type 2 diabetes is rising worldwide [1]. Several studies have shown social inequality in type 2 diabetes prevalence and outcome [2–4] where type 2 diabetes cluster in lower social groups [5]. Moreover, people with type 2 diabetes from lower social groups experience more complications, receive less care and live shorter lives than people with type 2 diabetes from higher social groups [6–11]. People with type 2 diabetes often suffer from other diseases besides diabetes, especially the socially disadvantaged [12, 13]. Type 2 diabetes care is extensive and consists of both regular clinic visits (consultations, biochemical tests etc.), self-care (e.g. dietary changes, exercise, medication adherence, foot care) and rehabilitation (e.g. patient education, exercise classes) directed at controlling blood sugar levels and other risk factors for complications and thereby minimize diabetes’ consequences on health [14]. Gender and family relations are related to how life is lived with type 2 diabetes and the resources that a patient with diabetes is able to mobilize to care for the disease [15–17].

Inequality in type 2 diabetes care and outcomes may be related to the work involved in accessing care and enactment of self-care, which may be difficult to prioritize or carry out when life is challenging [18–20]. The tasks involved in caring for type 2 diabetes (e.g. coordinating care, purchasing medicines, navigating services) constitute a burden of treatment risking to overwhelm patients, especially the socially vulnerable [17, 21, 22]. Reviewing research on health care access for vulnerable populations Dixon-Woods et al. (2006) have proposed a conceptual framework called “candidacy”. Candidacy describes how people’s eligibility for medical services is negotiated between the individual and the health services and how the conditions and context of people’s lives and the health services influence access to care and services [20]. Aspects of candidacy include *navigating* the health system knowing where to go with which health problem as well as *permeability* of services (*porous* services require fewer qualifications and less resources and effort to access).

Recently, social inequality in health and healthcare has gained political focus in Denmark [23]. National and regional type 2 diabetes strategies and programs mention the need to address social inequality in care [24, 25]. The national diabetes strategy promotes differentiating and customizing diabetes care to the individual patient’s needs and capacities as a means to enable everybody to benefit from care [25] and interventions aimed at supporting socially vulnerable type 2 diabetes patients access care are being implemented and evaluated [26]. In Denmark, five regions are responsible for health care provision and development of regional disease management programs inspired by the Chronic Care Model for the most prevalent diseases [27]. The disease management program for type 2 diabetes in the Capital Region of Denmark is characterized by an ideal of an active and informed patient able to mobilize resources and care for him- or herself. This ideal leads to some inherent expectations and a particular design of services. The program is designed for relatively well-educated and resourceful patients and the standardization of services itself favors patients who are able to meet the expectations ([28], p 32).

The ideal of active patients making choices about their own care, as the disease management program exemplify, permeate contemporary diabetes care [25, 28, 29]. The Dutch philosopher and ethnographer Annemarie Mol has argued that these ideals have the potential to displace previous care practices from future health care practice. Thus, we need to learn from what is already working in diabetes care. She argues that care is an important logic of diabetes care services, which ought not to be replaced by new public management and market logics. The ideal of good care is according to Mol silently incorporated into practice and does not argue for itself: Therefore, it needs to be studied and put into words [29]. Qualitative research is useful to “dig deeper” and explore and understand the complexities of caring for diabetes and generate hypotheses that may be investigated in larger samples using quantitative methods [30].

Standardization, IT-systems, quality assurance technologies and other organizational infrastructure influence clinical practice [31–33]. Standardization of clinical encounters and clinical trajectories are structuring the interaction between professionals and patients [32]. According to a Danish study from general practice, procedural standards for diabetes annual controls may lead to less patient-centered consultations and a focus on biochemical and biomedical results instead of the patient’s own perceived needs [32]. Rhodes et al (2006) have shown how a computerized checklist used in diabetes consultations influence the agenda and dialogue and leads to less patient centered care. Performance standards and indicators also shape the organizational context of diabetes care e.g. audit for quality assurance and benchmarking imposed by the regional government. Besides standardization, a logic of efficiency governs health care services and patients’ access to these. According to an ethnographic study of access to primary care services in Denmark, the sociopolitical context governing primary care favors efficiency. Professionals at the primary care clinics and their patients thus have to juggle the institutional logic of efficiency in order to provide/receive care that fits the need of the patient [34]. In an American ethnographic study of diabetes care, the authors argue that financial goals and managerial logics are prioritized above the needs of individual patients. While the North American and Danish health systems can hardly be compared, it is worth noticing how managerial logics with their efficiency and cost limiting goals transform clinical practice and targets of treatment in diabetes care [35]. Beedholm and Frederiksen have described four logics (the public management logic, the market logic, the medical profession logic and the care profession logic) that they see as co-existing and competing in Danish healthcare. In order to enhance patient involvement, they propose a fifth logic, the patient logic [36]. A recent Dutch study of long term care in private nursing homes argue that in practice different logics co-exist and are reconciled differently at different level of health care services and in different aspects of services [37].

In sum, different orders – standards, disease management programs, guidelines and strategies, representing logics of standardization and optimization seek to govern diabetes care practices. In order to understand how professionals may help socially vulnerable patients access and benefit from health care and perform the tasks diabetes care put on them we need to study not only these orders (care planning, strategies, standards and programs) but also how diabetes care unfolds in practice (specific interpersonal actions and actors).

In this study, we aim to describe what professionals at a specialized diabetes clinic do to adjust services to patient’s needs and make the tasks involved in diabetes care doable for socially vulnerable patients and how this work is embedded in an organizational and moral context. We seek to illustrate how the customization and differentiation of diabetes care is practiced at this particular clinic and with what consequences. To paraphrase Mol we aim to articulate the specificities of good care so we may talk about, and learn from, it [29].

## Methods

### Setting

Denmark has universal tax paid health care. People with uncomplicated type 2 diabetes receive care for their diabetes in general practice and may receive rehabilitation in their local municipality. People with type 2 diabetes who do not reach their treatment goals in general practice, who experience complications to their diabetes, or whose diabetes is difficult to control with standard medication are referred to specialized diabetes care by their general practitioner [28].

Data collection for the present study was carried out in an outpatient clinic specialized in endocrinology situated in a hospital in the Capital area of Denmark between February 2017 and March 2018. The clinic was chosen because the uptake area of the hospital is characterized as socio-economically deprived with a relatively high percentage of people with low education (25%) and an above region average of unemployment (13%) [38]. All parishes in the uptake area are categorized as very deprived related to housing, and some of the parishes as very deprived in relation to socioeconomic factors (education, income, occupation) [39]. This made the clinic experienced in caring for the socially vulnerable. When the clinic was contacted it became clear that providing good care for the socially vulnerable was also a priority for them and they therefore agreed to participate in the study,

### Participants

Sixteen patients with type 2 diabetes (9 male, 7 female, aged 35-73 years) and 12 professionals (7 doctors, 4 nurses, 1 secretary) working at the outpatient clinic participated in the study. The participating patients were recruited by nurses in the clinic. Nine of the patients were newly referred to the clinic and therefore did not have a relation to the recruiting nurses. Of the participating patients, three had no formal education after primary school. Most of the remaining had shorter vocational training. Three patients were in work rehabilitation, two of whom were at the time of the interviews on sick leave. Six patients were retired, three of them on early retirement. The remaining seven were employed. Characteristics of the participating patients are summarized in table 1. Of the seven doctors, five were senior doctors one of whom was head of the department and two were younger doctors finalizing their endocrinology specialization. Nurses had 2-20 years of experience in diabetes care. Three of the four nurses had long experience including several years at the diabetes outpatient clinic.

**Table 1:** Characteristics of participating patients

Characteristics of participating patients				
Pseudonym	Age	Sex	Education*	Employment status**
Torben	71	M	Short	Working
Klaus	60	M	Short	Sick leave
Yvonne	63	F	Short	Early retirement
Marianne	60	F	Short	Working
Rebecca	73	F	Short	Retired
Lars	58	M	Short	Working
Karsten	62	M	Short	Early retirement
Kalle	43	M	Middle	Working
Ulrik	35	M	Short	Working
Peter	58	M	Short	Working
Yussef	53	M	Short	Working
Monique	68	F	Short	Retired
Irene	58	F	Short	Work-rehabilitation
Fie	60	F	Middle	Sick-leave
Per	72	M	Short	Retired
Vibeke	64	F	Middle	Early retirement

*Education: short (no vocational training, semiskilled worker), middle (skilled worker, theoretical training less than three years), long (theoretical training more than 3 years)

**Employment status: work (normal conditions), work-rehabilitation (mandatory work-rehabilitation program to receive benefits), sick leave (receiving benefits but exempt from mandatory work-rehabilitation program), early retirement (retirement before the age of 65), retirement (retirement after the age of 65)

### Data collection

**Fig. 1:**
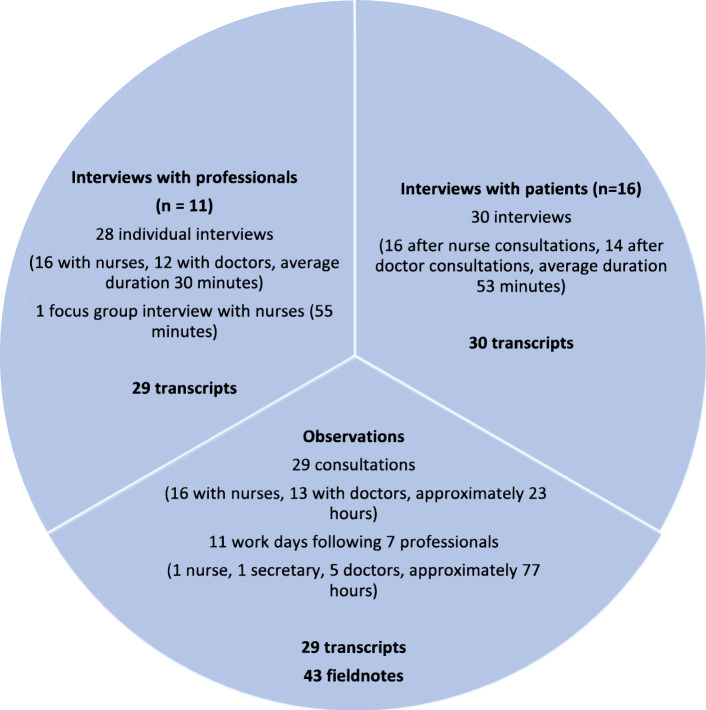
Data material collected

The data used for this article consist of 11 days of participant observation in the outpatient clinic and data on 16 patient trajectories (see Fig. 1). During participant observations professionals were shadowed during all work tasks including patient encounters. Some informal interviews also took place during participant observation. After participant observation elaborate field notes were written. For each patient trajectory, data consist of interviews with patient and providers as well as observations and transcripts of consultations (one consultation with a nurse, one subsequent consultation with a doctor). Three patients were not observed during their consultation with the doctor resulting in a total of 29 observed and transcribed consultations. After the consultations, the patient was interviewed about his/her experience of the encounter, trajectory, experiences with diabetes care and expectations of the services in- and outside the clinic. Patient interviews (30 interviews in total) were semi-structured and took place in the patient’s home, a separate room in the clinic or for one patient at the workplace of the interviewer. Patient interviews lasted 20-90 minutes (average 53 minutes), depending on the time the patient had available, and were transcribed verbatim. The participating professionals were interviewed after each encounter about their experience of the consultation, their impression of the patient and their expectations of the outcome of the patient’s trajectory as well as their experiences in working with socially vulnerable patients. Individual interviews with professionals were semi-structured, took place at the clinic, and lasted 15-40 minutes (average 30 minutes). We conducted one focus group interview with the four diabetes nurses lasting 55 minutes in order to gain insight into nurses’ experiences with patients too vulnerable to participate in the interview component of our study, the culture in the clinic, and organizational issues. Individual and focus group interviews with professionals (29 interviews in total) were transcribed verbatim.

In total, the data material consisted of 131 unique files (field notes, transcripts of clinical encounters and interviews). All of these were imported to NVivo 12 to maintain overview.

### Ethics

Before all interviews, the interviewer informed the patient or professional about the purpose of the study, anonymity and that the interviewee could withdraw his or her consent at any time or refuse to answer a question. All patients signed informed consent forms before participation. The study was approved by the Danish Data Protection Agency. According to Danish research guidelines, qualitative research requires no further approval. All names are pseudonyms. The size of the clinic and the anonymization of individual characteristics of patients ensures their anonymity. The professionals have pseudonyms and details about age and experience has been omitted to maintain their individual anonymity despite the recognizability of the clinic.

### Analysis

We did a stepwise analysis inspired by systematic text condensation [40]. Stepwise analysis involves starting analysis early in order to maintain the amount of data manageable [40]. During data collection, SR, AG, NT, GW and UC (authors not working in the clinic) met regularly to discuss the findings and analytical insights and to identify themes. When data collection finished SR wrote case descriptions for all included patient trajectories looking through all transcripts. In these two first steps we “moved from chaos to themes” ([40]: p 796). All field notes and all data concerning five trajectories selected to represent both male and female as well as working and not-working patients were inductively coded by SR. The same was done to the focus group interview with nurses. This amount of data was manageable and formed the second step in the analytical process moving from themes to codes ([40]: p 797). To validate this coding UC inductively coded all data about two of the patient trajectories and any disagreement about codes was discussed among SR, AG, NT, GW, and UC [41]. Authors SJ, IH and HP work (ed) at the outpatient clinic and were interviewed and observed during fieldwork. They therefore did not participate in the analytical process. SJ, IH and HP participated in the project formulation, recruited patients and commented on this manuscript as well as approved its final version.

## Theoretical framework

To understand how this diabetes clinic provides care for patients with type 2 diabetes we draw on Annemarie Mol’s conceptualization of “the logic of care” [29]. In her book, Mol distills a “logic of care” from the care practices that she and her colleges have observed in a Dutch hospital caring for (primarily type 1) diabetes patients. Mol uses the term ”logic” to explore *“what is appropriate or logical to do in some site or situation, and what is not.”* ([29]: p 9-10). In Mol’s account the “logic of care” is characterized by an ongoing adjustment of treatment and targets to the specific situation of an individual patient. In the logic of care, according to Mol, treatment is not linear, but involves ever-changing conditions, goals and values ([29]: p 61). This continuous adjustment involves experimenting, persistent and forgiving professionals that continue to care, even when things do not work out the way they were intended to [29]. Mol contrasts the logic of care with the logic of choice and its normative valuation of autonomy. In the logic of care, according to Mol, the patient’s need, not knowledge or preference, is the starting point for treatment ([29]: p 25). Inspired by Mol, we describe the logic of care in a particular diabetes outpatient clinic and combine this approach with the concept of permeability from the candidacy framework.

## Results

### A needs-based approach to treatment

A needs-based approach to care characterized clinical practice in our data from this outpatient clinic. This meant that both the timing and content of services were adjusted to fit the needs of the individual patient and that the clinicians approached the patients as persons with many needs – not only strictly related to diabetes.

### Adjustment of services: permeability, frequency, timing and content of consultations, and prescriptions

Professionals in the clinic adjusted the treatment and rehabilitation plans in several ways to meet the needs of the patient. Both the frequency of visits to the nurse, the scheduling and timing of visits and the content of the consultation were continuously adjusted to the individual patient. This customizing was based on an organizational flexibility and dedication from the individual professionals. However, as the nurses discussed in the focus group, this way of caring for patients was also part of the organizational culture in the clinic:

*“Well, it also has something to do with the approach here. Of course, it has to do with who we are as persons and as nurses. But it does also have something overall to do with how the approach is here.”* (Tove, nurse)

The clinic was open to patients in need, even when they did not have an appointment, though dropping in was not formally a possibility. This illustrated the permeability of the clinic. In the focus group interview with nurses, the following dialogue about patients dropping in took place:

*Tove (nurse): They just drop in for something. And it is rare, you know, that we send them out the door without anything.*

*Janne (nurse): Well it is also because we have our own patients, right. And that makes the patients, if all goes well, then they feel very safe with their nurse. And I think that is why it happens.*

The quotes show how the nurses felt a responsibility of attending to patients in need who dropped in without a scheduled appointment. This practice may be seen as part of a professional culture of meeting the patient’s needs, even if doing that does not fit with the organizational structure of an outpatient clinic.

The norm of helping patients in need, even if it takes some extra work or involves seeing patients without appointments, permeated the clinic all the way to the leading senior doctor:

*Nadim is not a patient at this clinic, but drops in on his way home from an x-ray in this hospital. He has been prescribed insulin for his type 2 diabetes at another specialized diabetes outpatient clinic, but experience trouble injecting it. Nadim is illiterate. Tove (nurse) goes to see Ib (leading senior doctor) in his office. They discuss what Tove should do and agree that she will contact the clinic where Nadim is seen to find out if they can deal with it.* (field note from participant observation)

Nurses in the clinic were able to schedule a follow-up visit sooner than standard if they and the patient thought there was a need for it or if the patient was worried and needed an extra consultation. This flexibility in frequency meant that the nurses were able to follow up and dose information to avoid overloading the patient. Nurses experienced that getting back to the same issue later or with another approach could help patients change behavior. The nurses during interviews also explained that *timing* was important and therefore they needed to be available with support once the patient was motivated for e.g. behavioral change.

Doctors were unable to follow-up on their diabetes patients as closely as they wished, which let to frustration and some doctors asking secretaries to add a specific patient to an already booked program. Often we observed delayed programs, skipped lunch breaks and very busy doctors. The nurses were busy as well and had full programs but were able to find time for their patients and able to adjust their programs to the need of the individual patient. If the nurse needed more time for a patient, she could book a double consultation without having to ask anyone:

*“You know that you should book half an hour, but sometimes you book one hour, right. Otherwise, it will not work out. [ … ] I mean we are supposed to proceed a little. On behalf of the patient. And survive the [work] day.”* (Freja, nurse)

The nurses appreciated the flexibility and independency that enabled them to organize their own work within some limits. Patients we interviewed experienced that the professionals in the clinic had time to attend properly to them. 58-year old Lars says: “Generally it seems like they have a bit more time. They don’t sit watching the clock when you are in a consultation.” Vibeke, who is a 64 year old retired teacher explains the importance of time and permeability in an interview:

Vibeke: “[They] always have time. Also, if something comes up. Just call.“

Interviewer: “Ok. What does that mean to you?”

Vibeke: “I feel safe”

The nurses and secretaries intended to schedule clinic visits on days of the week that suited the patient (e.g. related to homecare, work or other obligations). Some professionals even stretched their work schedule to meet the needs of the patients, e.g. came in early to see patients before regular clinic hours because the patient’s work only permitted him or her to attend the clinic before 8 am. Several professionals were also flexible with mutually adjusting programs so the patient could cluster appointments in order for clinic visits to interfere as little as possible with work obligations. The dedication of the individual professionals and the flexibility of the organization made it possible to adjust the appointment time to the patient’s everyday life.

While some examinations are usually scheduled for a specific visit (e.g. foot examination or urine test in annual control visits) professionals performed these examinations if the patient needed them, even if they were not planned for that day. Adjusting services was, according to the professionals, about getting to know the patient and his/her needs as well as being able to offer services at the “right moment”. This as one nurse expressed it, involved some “detective work”, in order for one to know a patient’s needs and preferences:

“*They come to us, and we are able to do it so much with focus on their needs. I actually believe that is how they prefer. They feel that we listen to them and do it in the way that they like the most, right*.”

(Freja, nurse)

Not only the timing, dosing and content of services was adjusted to the individual patient. Prescriptions were as well. Some patients need another treatment than standard in order for them to benefit from it. To illustrate this, we return to Nadim who dropped into the clinic because he needed help to inject his insulin. The situation described below illustrates that what constitutes an appropriate prescription depends not only on clinical guidelines and biomedical considerations but also on the patient’s life situation and resources.

*Tove has picked up an insulin pen for Nadim and is about to give it to him. She reminds Nadim that the pen has to be turned 20 times before injection. Nadim repeats. Tove shows him how to put on the needle and turn it up to 12 units [Nadim’s prescribed doses]. Nadim says he cannot see the numbers on the pen. He also confuses 12 and 21.*

(field notes from participant observation)

After Nadim had left the clinic, Tove and Janne (both diabetes nurses) discussed what had happened. They agreed that he would be able to learn to inject insulin, but with an insulin type that is easier to handle technically and only needs to be injected once daily.

In other situations, what constitutes an appropriate prescription depends on what the patient can afford. While services are offered for free in the clinic medicines and supplies are paid for partly by the patient.

In the consultation room, 60 year old Fie who is on benefits and currently on sick leave from work rehabilitation told her doctor Kristoffer, that she could not afford the medicine she had previously been prescribed. Kristoffer recognized that prescribing unaffordable medicine is not effective and deviated from clinical guidelines to meet Fie’s need for cheaper medication:

*“But I am aware that if one cannot afford the medicine, one does not take it and then it is of no use”* (Kristoffer, doctor)*.*

The nurses also experienced patients coming in who are unable to pay for their prescribed insulin – a very costly treatment. Both the patient’s low income (e.g. benefits) and the design of the reimbursement system made insulin too expensive for the patient to afford. In these cases, the nurses gave the patient an insulin pen or two until he or she again could afford it. The same happened with other supplies e.g. needles for blood sugar meters:

*Tove [nurse] asks him [patient] if he has any more needles. The patient says he uses them three times. Tove says he should not. They become bent. She gives him a box of needles.*

(Field note from participant observation)

In order for patients to benefit from their prescriptions, the nurses supported patients who (temporarily) could not pay themselves by giving medicine or supplies for free. In general, the municipality where the patient lives support test strips and needles for blood sugar measurement if they apply for it. As a standard, the diabetes nurses offered all new patients to write and send an application to the municipality for them. During the clinical encounter with the patient, they merely asked the patient to sign a predesigned application form making it as easy as possible for the patient to receive financial support for supplies. The systematization of applying for municipal support and maintaining an insulin and supply stock in the outpatient clinic, illustrate an organizational structure that enables helping patients with low economic resources.

While professionals were able to adjust prescriptions there were also situations where they were unable to make medication and patient meet. During participant observation with a doctor a middle-aged man working as a bus driver came into the consultation. His blood sugar was too high, and the doctor Ditte wanted to prescribe blood sugar lowering injections. The patient rejected this prescription as his wife had experienced side effects of the same medication. The following is a field note exempt from the consultation:

*Ditte makes it clear to him, that next step is insulin, saying that she assumes he does not want that due to his driver’s license. It almost seems like a threat. She also tells him that if he is to continue in the clinic, he must be willing to do some of the things the clinic suggests, otherwise there is no reason for him not to be cared for by his general practitioner.* (field note of participant observation)

After the consultation Ditte was clearly frustrated and provoked by the patient’s passivity. When unable to fulfil her own ambition of improving the patient’s disease, and collaborate with the patient, Ditte turned to shocking him instead, something reported elsewhere [42]. The episode also illustrates the organizational logic to spend resources rationally and discharge patients taking up scarce resources (consultations) if there is no (or not enough) perceived benefit. The economic and organizational demand to discharge patients was also mentioned several times by doctors when deciding whether a patient should continue to be cared for in the clinic:

*Ditte tells me that she preferably should discharge two patients a day on average, at least. In practice she says that some days its none and some days it is five. But she still has it in mind.*

(field note from participant observation)

Adjusting agendas, frequency of visits, examinations and prescriptions to the need and conditions of the patient illustrates how treatment in the clinic was “attuned to everything else” ([29]: p 61). It also illustrates that in this logic of care, what is the best choice from the medical perspective (represented in treatment guidelines) is not necessarily the best choice for the patient, considering his or her specific situation. Even if the services are adjusted and flexible the patients in the study experienced that services are structured (for them) in the clinic. Many of them appreciated that and saw it as a burden lifted from their shoulders that they were not in charge of scheduling appointments. Vibeke found comfort in “always knowing what will happen in the clinic” and Yussef appreciated that the staff printed his record and lab results and explained to him, what they meant. It made him feel safe he said. The participating patients also found it reassuring to have their diabetes been taken care of by specialized staff.

### A person with many needs: respecting other needs and helping the patient to become a candidate for services elsewhere

While the patients in this study are referred to the clinic with diabetes, many of them experienced other problems, medical as well as social. As mentioned above, the agenda for a consultation may be changed based on the needs of the patient. If other needs or issues were consuming the patient’s energy or time, if the patient was worried about symptoms or family members or was in the midst of crisis, the professionals embraced these issues in the conversation. When patients worried about other symptoms not strictly related to diabetes the nurse took time to hear about it and helped the patient handle it e.g. by asking a doctor in one of the other consultation rooms or guiding the patient to where he or she could get help with it.

In the focus group interview, the nurses described how they approach patients with many needs:

*Freja: “Really, they must feel that we listen to them. So, you may find yourself working with things, where you think, well, that was not the plan, but we will have to in order to move on.”*

[ … ]

*Rie: “Sometimes you need a longer consultation. Because so many other things appear on the way. And if you don’t attend to these simultaneously, they may not feel that you care for them. Or that they trust you.”*

Some patients also needed help to get on further in the health system in order to benefit from services or referrals. The professionals often helped patients with this. Either by guiding them in the right direction or by negotiating access to services, thus making other services more permeable.

The patients’ feet were checked once yearly at the clinic and if needed the patient was offered a referral to chiropody elsewhere. In order for patients to benefit from their referral, diabetes nurse Rie printed a list with contact details of state-authorized chiropodists serving close to the patient’s home. When asked why she did that, Rie answered:

*“Because it is my experience that it is a bit beyond them to look for chiropodists. Especially because not all chiropodists are authorized. Then they don’t receive the referrals. It is much easier then, if I just print out something where they have a phone number and an address to respond to.”* (Rie, nurse)

If referrals for one reason or another did not result in the patient receiving a service elsewhere (e.g. in a cardiology department or municipal rehabilitation) the nurses double checked referrals, resent the referral or called the other department on behalf of the patient, something often observed during participant observation.

Sometimes professionals actively engaged in negotiating access to services for the patient. This is exemplified with Nadim who dropped into the clinic. Tove put a lot of effort into contacting the clinic where Nadim was usually seen for his diabetes. Negotiating access for him there involved navigating through a secretary and a key-yourself system to a nurse and convincing the nurse of Nadim’s need. Tove experienced that she had to pressure the nurse at the other clinic to give Nadim the care Tove believed he needed. After talking to the other hospital, Tove prepares Nadim to go to Hospital B:

*Tove says that she talked to Hospital B and that he should go there now and get help with the insulin. Nadim says they know him there. Tove tells Nadim to ask for a home care nurse that can help him to inject the insulin in the beginning. She repeats “home care nurse” clearly. Tove has written the department number at Hospital B on a post-it.*

(field notes from participant observation)

Tove prepared Nadim so that he could get the services he needs to benefit from his prescription. The small things of making a note and telling Nadim to ask for what he needs (a home care nurse) illustrate the specificity of helping an individual patient to benefit from health services. It also illustrates how practicing the logic of care in the clinic involved doing much more than sticking to guidelines and letting the patient do his access work himself. In the logic of care, the professional steps in where the patient needs help – be it calling, negotiating, injecting, writing notes or arming him with the right words to express his/her need elsewhere. The professionals helped the person to become a - with Dixon-Woods et al.’s word - *candidate* for the specific service that he or she needed through supporting the patient to navigate the health services and negotiate access to them [20].

## Discussion

In our analysis, we have argued that the logic and practice of care in this particular clinic are characterized by a needs-adjusted approach to both access, timing, and content of consultations as well as attending to needs not directly related to diabetes. This logic of care permeates the clinic all the way to the leading senior doctor and is mirrored in the organizational conditions of clinical practice. From studying the care practices and the normative and organizational conditions under which these take place, we have described a logic of care with patients’ multiple needs at its center.

According to a recent Danish study, socially vulnerable patients with type 2 diabetes prefer patient-centred individualized and needs adjusted self-management support [43]. Several reviews trying to integrate previous definitions of patient-centred care include a focus on patients’ needs as an important element of patient-centred care [44, 45]. Castro et al (2016) highlight the connection between patient-centered care and individualization of care and argue that an important attribute of patient-centeredness is seeing the patient as a unique person and trying to meet the “specific needs, values and beliefs of patients.” ([45]: 1929). In our study, we have illustrated how this happens in practice with all the particularities of individual patients’ lives and individualized solutions and efforts of professionals. How it involves continuously adjusting schedules, agendas, expectations, medications etc. to what the patient needs. This illustrates that practicing care centered on the patient’s needs is not a matter of implementing a new tool, but of an organizational culture and organizational flexibility enabling the professionals to achieve needs-based care in practice. In this way our study may be said to exemplify in diabetes care what Öhlén et al. have described in person-centered palliative care, namely that it is “a way of being in the professional world”, not a range of tools [46]. Insights about culturally and contextually appropriate diabetes interventions gained from qualitative research in selected clinics in India have subsequently been tested in a clinical trial showing effect on primary outcomes [47, 48]. Likewise, implementing organizational conditions flexible enough for individualization of care and nourishing an including culture in clinics may also be tested to see if what works in this clinic can also work elsewhere.

Beedholm and Frederiksen have argued that implementing an ideal of patient involvement is challenged by cultural and structural factors in the form of institutional logics already existing in the clinic [36]. In our analysis, we have illustrated the local logic of care practiced in this clinic despite the existence of standardization and efficiency logics and technologies. We have shown that in practice focusing on the individual patient’s needs is possible with an organization and culture enabling it. An American ethnographic study of hospital-based diabetes care concluded that professional expertise and concern for the individual patient’s wellbeing was overruled by managerial, organizational and market logics’ focus on efficiency and profitability. This led to professionals experiencing inability to meet the patient’s needs within the frame of what the insurance company allows [35]. While the market logic dominates in their account, they also provide examples of how clinicians, as the clinicians in our study, worked around existing limitations of access to medicines [35].

Previous studies have noticed the importance of the organizational culture in the clinic [45, 49]. An American experimental study found a statistically significant association between medical practice culture and the participating general practitioners’ decision-making and management of diabetes. Especially collegiality, information emphasis and organizational trust were positively related to performing necessary examinations [49]. In our analysis, the importance of culture in the clinic is exemplified as the normative conditions and the acceptance of putting aside demands of e.g. efficiency or clinical guidelines for medication prescription in order to be able to meet the needs of the patient.

Some limitations of this research are worth noting. Data collection was limited to one specialized clinic making it impossible to conclude on care logics and practices elsewhere. However, focusing fieldwork on one site enabled us to deepen our understanding of practice in this particular place. Thus, the study has similarities with the qualitative case study which was appropriate because this empirical approach made it possible to capture the actual care practice “within its real-life context” ([50]: p 18). Through our “thick description” of care practices in this clinic researchers elsewhere knowing their particular context may consider the transferability of our findings [51]. Another limitation is that participating patients are not among the most socially vulnerable patients as these were hard to recruit due to the fragility of their relation to the clinic. This challenge is well known and has been reported elsewhere [52]. We tried to address this through asking all professionals about their experiences with other (more) socially vulnerable patients and through addressing the issues of caring for the most vulnerable patients during the focus group interview. During participant observation we also witnessed consultations with an unselected group of patients receiving care in this clinic, some of whom were much more socially and physically vulnerable than the 16 interviewed patients. While nurses recruited the interviewed patients, 7 of whom were previously known to nurses, which holds the possibility of introducing selection bias, this was not the case during participant observation. The interviewer also stated before interviews with each patient, that she was not employed at the clinic and that the interview would have no consequences for the patient’s health care.

Through combining participant observation and interviews, it has been possible to grasp both diabetes care as it is practiced in the everyday work of clinicians as well as clinicians’ and patients’ perception of this practice and its consequences. Participant observation made it possible to observe care practices and situations that were unlikely to have been revealed through interviews alone and interviews enabled us to understand the meaning of practice for the involved actors and to broaden our understanding of clinical practice beyond what we observed being physically present. Our stepwise analysis and continuous engagement with the material during data collection has strengthened our empirical base, analytical insights, and the credibility of our research [40, 51].

## Conclusion

In this article we have argued that practicing a need-based approach to treatment involves a continuous adjustment of permeability, frequency, timing and content of consultations, as well as prescriptions in order to meet the individual patient’s need. It also involves attending to patient needs not directly related to diabetes and preparing the patient to negotiate access to services outside the clinic that the patient needs. Thus, helping the patient to become a candidate for care in other health care settings. This needs-based approach to treatment is in this specific case enabled by an organization flexibility, an organizational culture and dedicated professionals. Taking findings from this study elsewhere would involve creating the conditions for organizational flexibility, nourishing an organizational culture honouring patient needs and recognizing the efforts of dedicated professionals who every day do small things that enable patients to access and benefit from the resources put into the health services.

## Data Availability

The datasets generated and analysed during the current study are not publicly available due to the promised confidentiality and anonymity of the informants but are available from the corresponding author on reasonable request.

## References

[1] WHO. Diabetes [Internet]. 2019 [cited 2020 Jan 27]. Available from: https://www.who.int/news-room/fact-sheets/detail/diabetes

[2] Dinca-Panaitescu S, Dinca-Panaitescu M, Bryant T, Daiski I, Pilkington B, Raphael D. Diabetes prevalence and income: Results of the Canadian Community Health Survey. Health Policy (New York) [Internet]. 2011;99(2):116–123. Available from: 10.1016/j.healthpol.2010.07.018 10.1016/j.healthpol.2010.07.018 20724018

[3] Agardh E, Allebeck P, Hallqvist J, Moradi T, Sidorchuk A (2011). Type 2 diabetes incidence and socio-economic position: A systematic review and meta-analysis. Int J Epidemiol.

[4] Espelt A, Arriola L, Borrell C, Larranaga I, Sandin M, Escolar-Pujolar A (2011). Socioeconomic Position and Type 2 Diabetes Mellitus in Europe 1999- 2009: a Panorama of Inequalities. Curr Diabetes Rev [Internet].

[5] Candib L (2007). Obesity and Diabetes in Vulnerable Populations: Reflection on Proximal and Distal Causes. Ann Fam Med.

[6] Sortsø C, Lauridsen J, Emneus M, Green A, Jensen PB (2018). Social inequality in diabetes patients’ morbidity patterns from diagnosis to death - A Danish register-based investigation. Scand J Public Health.

[7] Sortsø C, Lauridsen J, Emneus M, Green A, Jensen PB (2017). Socioeconomic inequality of diabetes patients’ health care utilization in Denmark. Health Econ Rev.

[8] Forssas E, Keskimäki I, Reunanen A, Koskinen S. Widening socioeconomic mortality disparity among diabetic people in Finland. Eur J Public Health. 2003;13(1):38–43. 10.1093/eurpub/13.1.38 12678312

[9] Ricci-Cabello I, Ruiz-Pérez I, De Labry-Lima AO, Márquez-Calderón S (2010). Do social inequalities exist in terms of the prevention, diagnosis, treatment, control and monitoring of diabetes? A systematic review. Heal Soc Care Community.

[10] Holm AL, Andersen GS, Jørgensen ME, Diderichsen F (2018). Is the rule of halves framework relevant for diabetes care in Copenhagen today? A register-based cross-sectional study. BMJ Open..

[11] Heltberg A, Andersen JS, Kragstrup J, Siersma V, Sandholdt H, Ellervik C (2017). Social disparities in diabetes care: a general population study in Denmark. Scand J Prim Health Care..

[12] Schiøtz ML, Stockmarr A, Høst D, Glümer C, Frølich A (2017). Social disparities in the prevalence of multimorbidity - A register-based population study. BMC Public Health..

[13] Barnett K, Mercer SW, Norbury M, Watt G, Wyke S, Guthrie B (2012). Epidemiology of multimorbidity and implications for health care, research, and medical education: A cross-sectional study. Lancet [Internet]..

[14] The Danish Diabetes Association [Diabetesforeningen]. Tablet treatment [Tabletbehandling] [Internet]. 2018 [cited 2020 Feb 3]. Available from: https://diabetes.dk/diabetes-2/fakta-om-diabetes-2/behandlingskvalitet/tabletbehandling.aspx

[15] Sriram V, Sridhar G, Madhu K (2001). Gender differences in living with type 2 diabetes. Int J Diab Dev Ctries.

[16] Sridhar G (2020). On Psychology and Psychiatry in Diabetes. Indian J Endocrinol Metab..

[17] Serrano V, Spencer-Bonilla G, Boehmer K, Montori V. Minimally Disruptive Medicine for Patients with Diabetes. Curr Diab Rep. 2017;17. 10.1007/s11892-017-0935-7 28942581

[18] Nettleton S (2013). The Sociology of Health and Illness.

[19] Hinder S, Greenhalgh T (2012). “This does my head in”. Ethnographic study of self-management by people with diabetes. BMC Health Serv Res.

[20] Dixon-Woods M, Cavers D, Agarwal S, Annandale E, Arthur A, Harvey J (2006). Conducting a critical interpretive synthesis of the literature on access to healthcare by vulnerable groups. BMC Med Res Methodol.

[21] Sav A, Salehi A, Mair FS, McMillan SS (2017). Measuring the burden of treatment for chronic disease: Implications of a scoping review of the literature. BMC Med Res Methodol..

[22] Pesantes M, Tetens A, Del Valle A, Miranda J (2019). “It is Not Easy Living with This Illness”: A Syndemic Approach to Medication Adherence and Lifestyle Change among Low-income Diabetes Patients in Lima, Peru. Hum Organ..

[23] Udesen CH, Skaarup C, Petersen MNS, Ersbøll AK. Social ulighed i sundhed og sygdom: Udviklingen i Danmark i perioden 2010-2017 [Internet]. København; 2020. Available from: https://www.sst.dk/-/media/Udgivelser/2020/Ulighed-i-sundhed/Social-ulighed-i-sundhed-og-sygdom-tilgaengelig.ashx

[24] The Capital Region [Region Hovedstaden]. Disease Management Program for Type 2 Diabetes [Forløbsprogram for type 2 diabetes] [Internet]. 2019. Available from: https://www.regionh.dk/til-fagfolk/Sundhed/Tvaersektorielt-samarbejde/kronisk-sygdom/Forløbsprogrammer/Documents/Revideret FP for T2DM Maj 2019.pdf

[25] Ministry of Health. Den nationale diabeteshandlingsplan (The national diabetes strategy) [Internet]. Sundheds- og ældreministeriet (The Ministry of Health and Elderly); 2017. Available from: https://www.sum.dk/~/media/Filer - Publikationer_i_pdf/2017/Den-Nationale-Diabetes-Handlingsplan/2National diabeteshandlingsplan.pdf

[26] Innovationsfonden. Investeringer [Internet]. Investeringsoversigt 2018. 2020 [cited 2020 Nov 26]. Available from: https://innovationsfonden.dk/da/investeringer/investeringsoversigt?field_program_target_id=&field_start_year_value=2018&field_region_target_id=50&field_area_target_id=37&page=7

[27] Frølich A, Jacobsen R, Knai C. Denmark. In: Nolte E, Knai C, editors. Assessing chronic disease management in European health systems: Country reports [Internet]. European Observatory on Health Systems and Policies; 2015. p. 17–25. Available from: https://www.euro.who.int/__data/assets/pdf_file/0009/270729/Assessing-chronic-disease-management-in-European-health-systems.pdf 29035490

[28] Region Hovedstaden. Forløbsprogram for Type 2 diabetes [Internet]. København; 2016. p. 1–43. Available from: https://www.regionh.dk/til-fagfolk/Sundhed/Tvaersektorielt-samarbejde/kronisk-sygdom/Forløbsprogrammer/Documents/Revideret FP for T2DM Maj 2019.pdf

[29] Mol A (2008). The Logic of Care: Health and the problem of patient choice.

[30] Ritholz MD, Beverly EA, Weinger K (2011). Digging deeper: The role of qualitative research in behavioral diabetes. Curr Diab Rep..

[31] Rhodes P, Langdon M, Rowley E, Wright J, Small N (2006). What does the use of a computerized checklist mean for patient-centered care? The example of a routine diabetes review. Qual Health Res..

[32] Lippert ML, Reventlow S, Kousgaard MB (2017). The uses and implications of standards in general practice consultations. Heal (United Kingdom)..

[33] Timmermans S, Berg M (2003). The Gold Standard: The Challenge of Evidence-based Medicine and Standardization in Health Care.

[34] Andersen RS, Vedsted P (2015). Juggling efficiency. An ethnographic study exploring healthcare seeking practices and institutional logics in Danish primary care settings. Soc Sci Med..

[35] Hunt LM, Bell HS, Martinez-Hume AC, Odumosu F, Howard HA (2019). Corporate Logic in Clinical Care: The Case of Diabetes Management. Med Anthropol Q..

[36] Beedholm K, Frederiksen K (2019). Patient involvement and institutional logics: A discussion paper. Nurs Philos.

[37] Kruse FM, Ligtenberg WMR, Oerlemans AJM, Groenewoud S, Jeurissen PPT (2020). How the logics of the market, bureaucracy, professionalism and care are reconciled in practice: an empirical ethics approach. BMC Health Serv Res..

[38] Lau C, Lykke M, Andreasen A, Bekker-Jeppesen M, Buhelt L, Robinson K, et al. Health Profile 2013 - Chronic Disease [Sundhedsprofil 2013 - Kronisk sygdom] [Internet]. Copenhagen; 2015. Available from: https://www.regionh.dk/presse-og-nyt/pressemeddelelser-og-nyheder/Documents/Sundhedsprofil_RegionHovedstaden2013_Kronisk_sygdom_sammenfatning.pdf

[39] Meijer M, Engholm G, Grittner U, Bloomfield K (2013). A socioeconomic deprivation index for small areas in Denmark. Scand J Public Health..

[40] Malterud K (2012). Systematic text condensation: A strategy for qualitative analysis. Scand J Public Health..

[41] Tong A, Sainsbury P, Craig J (2007). Consolidated criteria for reporting qualitative research (COREQ): A 32-item checklist for interviews and focus groups. Int J Qual Heal Care..

[42] Wens J, Vermeire E, Van Royen P, Sabbe B, Denekens J (2005). GPs’ perspectives of type 2 diabetes patients’ adherence to treatment: A qualitative analysis of barriers and solutions. BMC Fam Pract..

[43] Christensen NI, Drejer S, Burns K, Lundstrøm SL, Hempler NF (2020). A qualitative exploration of facilitators and barriers for diabetes self-management behaviors among persons with type 2 diabetes from a socially disadvantaged area. Patient Prefer Adherence..

[44] Scholl I, Zill JM, Härter M, Dirmaier J (2014). An integrative model of patient-centeredness-A systematic review and concept analysis. PLoS One.

[45] Castro EM, Van Regenmortel T, Vanhaecht K, Sermeus W, Van Hecke A. Patient empowerment, patient participation and patient-centeredness in hospital care: A concept analysis based on a literature review. Patient Educ Couns [Internet]. 2016;99(12):1923–1939. Available from: 10.1016/j.pec.2016.07.026 10.1016/j.pec.2016.07.026 27450481

[46] Öhlen J, Reimer-Kirkham S, Astle B, Håkonson C, Lee J, Eriksson M (2017). Person-centred care dialectics - Inquired in the context of palliative care. Nurs Philos..

[47] Johnson LCM, Chwastiak L, Poongothai S, Tandon N, Anjana RM, Aravind S (2018). Adaptations and patient responses to behavioral intervention components in a depression-focused chronic disease care model implemented in India. Transl Behav Med..

[48] Ali MK, Chwastiak L, Poongothai S, Emmert-Fees KMF, Patel SA, Anjana RM (2020). Effect of a Collaborative Care Model on Depressive Symptoms and Glycated Hemoglobin, Blood Pressure, and Serum Cholesterol among Patients with Depression and Diabetes in India: The INDEPENDENT Randomized Clinical Trial. JAMA.

[49] Shackelton R, Link C, Marceau L, Mckinlay J (2009). Does the culture of a medical practice affect the clinical management of diabetes by primary care providers?. J Heal Serv Res Policy.

[50] Yin RK (2003). Case study research: design and methods.

[51] Lincoln YS, Guba EG (1985). Naturalistic Inquiry.

[52] Sydor A (2013). Conducting research into hidden or hard-to-reach populations. Nurse Res..

